# Hydrophobic Cu_2_O Quantum Dots Enabled by Surfactant Modification as Top Hole‐Transport Materials for Efficient Perovskite Solar Cells

**DOI:** 10.1002/advs.201801169

**Published:** 2019-02-07

**Authors:** Chang Liu, Xianyong Zhou, Shuming Chen, Xingzhong Zhao, Songyuan Dai, Baomin Xu

**Affiliations:** ^1^ SUSTech Academy for Advanced Interdisciplinary Studies Southern University of Science and Technology Shenzhen Guangdong Province 518055 China; ^2^ Department of Materials Science and Engineering Southern University of Science and Technology Shenzhen Guangdong Province 518055 China; ^3^ Department of Electrical and Electronic Engineering Southern University of Science and Technology Shenzhen Guangdong Province 518055 China; ^4^ Department of Physics Wuhan University Wuhan Hubei Province 430072 China; ^5^ Dean of Renewable Energy School North China Electric Power University Beijing 102206 China

**Keywords:** inorganic interfacial layer, perovskite solar cells, quantum dots, surface modification

## Abstract

The utilization of an inorganic hole‐transport layer (HTL) is one of the most effective methods to improve the stability and reduce the cost of perovskite solar cells (PSCs). However, achieving high‐quality inorganic HTL films, especially HTL films in n‐i‐p structures, via solution processes remains a big challenge. Here, a simple surface modification strategy for low‐cost and stable cuprous oxide (Cu_2_O) quantum dots is proposed, which utilizes a silane coupling agent. The modified Cu_2_O can be directly deposited on the perovskite film as the top HTL without decomposing the perovskite to maintain an n‐i‐p structure. The efficiency (18.9%) of PSCs with surface‐modified Cu_2_O as the HTL is significantly higher than that (11.9%) of PSCs with unmodified Cu_2_O, which is also the record efficiency for a Cu_2_O‐based perovskite solar cell in n‐i‐p structure. The enhanced performance of PSCs is attributed to the remarkably enhanced film properties achieved through surface modification. Moreover, because of the dopant‐free technology and hydrophobic surface, the Cu_2_O‐based PSCs have distinctly better stability than 2,2′,7,7′‐tetrakis[*N*,*N*‐di(4‐methoxyphenyl)amino]‐9,9′‐spiro‐bifluorene‐based PSCs.

Perovskite solar cells (PSCs) have received significant research interest as effective photovoltaic materials due to their high solar conversion efficiencies and low cost.^1^, ^2^, ^3^ In the past 8 years, the efficiencies of PSCs have increased from 3.8% to over 22.1%.^4^, ^5^, ^6^, ^7^ To date, hole‐transport materials (HTMs) have been recognized as a crucial factor for constructing efficient PSCs, especially for n‐i‐p‐type PSCs.^8^, ^9^, ^10^, ^11^ Most high‐efficiency PSCs (PCE > 21%) with an n‐i‐p structure were configured by using TiO_2_ as an electron‐transport material (ETM) and 2,2′,7,7′‐tetrakis[*N*,*N*‐di(4‐methoxyphenyl)amino]‐9,9′‐spiro‐bifluorene (Spiro‐OMeTAD) or poly[bis(4‐phenyl)(2,4,6‐trimethylphenyl)amine] as the HTM. However, the high cost of these HTMs has severely limited the large‐scale production of PSCs. Moreover, to improve the hole mobility of organic HTMs, water‐absorbing additives such as Li‐bis(trifluoromethanesulfonyl) imide (Li‐TFSI) have been widely introduced as dopants; these additives increase the moisture absorption and decomposition of the perovskite layer.^12^, ^13^, ^14^, ^15^ Therefore, developing low‐cost and dopant‐free inorganic HTMs is an important approach to improve the stability and reduce the cost of PSCs.^16^ To date, owing to their excellent stability, high hole mobility, and low cost, several metal oxides and compounds (such as NiO, MoO_3_, CuSCN, and CuI) have been successfully employed to replace organic HTMs in perovskite devices.^17^, ^18^, ^19^ Research on NiO*_x_*‐based PSCs with inverted planar structure has achieved great progress.^20^, ^21^ Cu‐based HTMs, such as CuI and CuSCN, have attracted much attention for the preparation of PSCs.^22^, ^23^, ^24^ However, it should be noted that most inorganic HTM‐based PSCs with high efficiency were only achieved in inverted p‐i‐n structures, which requires expensive ETMs such as [6,6]‐phenyl C61 butyric acid methyl ester or [6,6]‐phenyl C71 butyric acid methyl ester.^25^, ^26^, ^27^ Hence, for the fabrication of high‐efficiency and low‐cost PSCs, devices with an n‐i‐p structure appear to be more promising because stable and inexpensive ETMs such as TiO_2_, ZnO, and SnO_2_ can be used.^28^, ^29^, ^30^ Until now, the PCE of PSCs based on inorganic HTMs with an n‐i‐p structure was still far below that of classical devices using organic HTMs because a low‐cost, solution‐processing method for directly depositing HTMs on the surface of the perovskite layer in n‐i‐p‐type PSCs has yet to be developed. The deposition of HTMs is hampered by the high annealing temperatures and polar solvents, which could quickly degrade the perovskite layer. Hence, a general and facile approach to fabricating inorganic HTM‐based, efficient and stable PSCs with an n‐i‐p structure is urgently needed.

Spin‐coating with a nanoparticle dispersion solution is a feasible solution‐process method to deposit high‐quality films.^31^ For n‐i‐p structures, nonpolar reagents, such as 2‐methylanisole, chlorobenzene, or toluene, are normally selected as dispersion solvents to prevent perovskite damage.^32^ However, due to their naturally hydrophilic surface, most inorganic nanoparticles are hardly dispersed in nonpolar solvents. To improve nanoparticle dispersion in nonpolar solvents, the utilization of silane coupling agents for the surface modification of nanoparticles is an effective strategy that has been demonstrated in other fields, indicating that nonpolar inorganic nanoparticle dispersions can be prepared via surface modification with silane coupling agents.^33^, ^34^, ^35^

Herein, we propose a simple surface modification approach to improve the dispersion of inorganic nanoparticles in nonpolar solvents by taking cuprous oxide (Cu_2_O) quantum dots (QDs) as an example. Cu_2_O, a narrow‐band‐gap semiconductor (e.g., 2.1–2.2 eV), is a promising inorganic HTM due to its low electron affinity and very high hole mobility.^36^, ^37^, ^38^ Previous studies have indicated that Cu_2_O is an excellent inorganic HTM and may lead to a higher theoretical PCE than NiO*_x_*, CuSCN, CuI, and Spiro‐OMeTAD.^39^, ^40^ The over 19% efficiency of CuO*_x_*‐based PSCs has been achieved in p‐i‐n structure. However, this structure still suffers a competitive light absorption in the range below 550 nm from CuO*_x_* and the utilization of expensive [6,6]‐phenyl‐C61‐butyric acid methyl ester (PCBM). In this work, Cu_2_O QDs were synthesized in aqueous solution by a low‐temperature redox reaction.^41^ Because the as‐prepared Cu_2_O QDs were coated with numerous hydrophilic hydroxyls and thus hardly dispersed in nonpolar solvent, several commercial silane coupling agents were employed to modify the Cu_2_O surface. Notably, the functional group of the silane coupling agent has a great effect on the properties of the modified Cu_2_O QDs. After carefully investigating the different effects of silane coupling agents on the Cu_2_O QD‐based PSCs, ethenyltriethyloxysilane was found to be the best surface modification agent for Cu_2_O. The appropriate surface modification not only led to better dispersion of Cu_2_O in nonpolar solvent but also introduced a hydrophobic group on the Cu_2_O surface, which further formatted the moisture protection layer in PSCs. Based on this design, a compact, hydrophobic, inorganic HTM layer was fabricated, and both the efficiency and the stability of PSCs were enhanced by the presence of this layer. To verify this performance, we used Cu_2_O QDs as inorganic HTMs to fabricate compact TiO_2_/mesoporous TiO_2_ (mp‐TiO_2_)/perovskite/Cu_2_O/Au solar cells. As expected, the PSCs based on the Cu_2_O HTM exhibited excellent power conversion efficiency and high stability under long‐term illumination. Due to the high film quality of the HTM layer, a champion PCE of 18.9% was achieved by employing modified Cu_2_O as the HTM layer, representing an extremely high enhancement compared with that of unmodified Cu_2_O‐based PSCs (11.9%). Moreover, the PSCs prepared by this strategy could maintain over 90% of their initial efficiency after 30 d when stored in ambient air without encapsulation. Because this enormously enhanced performance of PSCs as a result of surface modification is universally applicable to any inorganic HTM‐based PSC, this new mechanism and approach established for PSCs could lead to a significant advance in the field toward practical PSC applications.

The solar cell configuration and the energy‐level diagram of the materials used are shown in **Figure** **1**. As shown in Figure 1a, the commercially available fluorine‐doped tin oxide (FTO) glass substrate was first covered with a thin (≈30 nm) TiO_2_ compact layer as the hole‐blocking layer before spin‐coating with mesoporous TiO_2_ paste to form a mesoporous TiO_2_ film (electron‐extracting scaffold). The light harvester, Cs_0.05_FA_0.81_MA_0.14_PbI_2.55_Br_0.45_ (denoted as CsFAMA), was then introduced using a reported one‐step deposition method, where the perovskite solution was spin‐coated on TiO_2_ and chlorobenzene was used as an anti‐solvent. After annealing, the Cu_2_O QD dispersion was introduced by the spin‐coating technique to ensure effective hole extraction and collection at the Au cathode. We plotted the energy‐level diagram of the materials used in our devices in Figure 1b according to the positions of the conduction band‐edge and valence band‐edge (VB) of the Cu_2_O QDs. It should be noted that the energy band level of Cu_2_O QDs before and after surface modification did not exhibit obvious difference (Figure S1 and Table S1, Supporting Information). This result demonstrates that the amount of modification agent coated on the surface of Cu_2_O QDs is very small. As shown in Figure 1b, the VB of the Cu_2_O QDs (−5.28 eV) in our study is similar to the VB of CsFAMA (−5.60 eV), which favors the transfer of holes from the perovskite layer into the inorganic HTM.^42^ Due to the high hydrophilic surface of Cu_2_O QDs, the typically used nonpolar solvents cannot provide a reasonable dispersity of Cu_2_O QDs. Therefore, the challenge in solution processing an inorganic material onto perovskite is attaining sufficient dispersion of Cu_2_O QDs in a suitable solvent that does not dissolve the perovskite material. In our work, the silane coupling agent ethenyltriethyloxysilane was introduced as a surface‐modifying agent to improve the dispersion of Cu_2_O QDs in nonpolar solvent. The Cu_2_O QDs were functionalized in situ and became hydrophobic as the silane molecules reacted to form covalent bonds on the surface of the Cu_2_O QDs. As a result, the surface‐modified Cu_2_O QDs could be well dispersed in 2‐methylanisole, which led to the formation of a high‐quality film as the HTM layer (Figure 1c).

**Figure 1:**
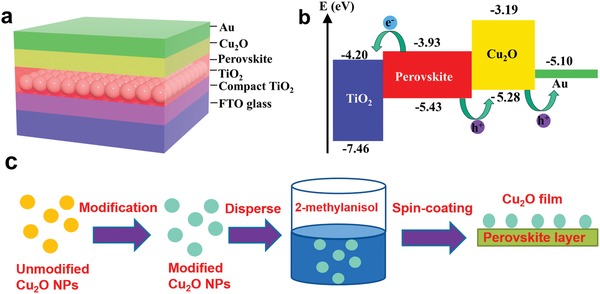
Device architecture and energy‐level diagram. a) Schematic view of the PSC configuration: FTO glass, compact TiO_2_ underlayer, mesoporous TiO_2_ with infiltrated perovskite, Cu_2_O HTM, and Au layer. b) Energy‐level diagram of the TiO_2_/perovskite/Cu_2_O/Au device showing electron injection and hole extraction. c) Schematic illustration of the deposition process of the Cu_2_O film.

Importantly, the functional group of the silane coupling agent has a great effect on the properties of the modified Cu_2_O QDs. To investigate this effect, three silane coupling agents were tested as the surface modification agent for the Cu_2_O QDs. The surface modification mechanism of the silane coupling agent on the Cu_2_O QDs is shown in **Figure** **2**d. As shown in Figure 2a–c, the three silane coupling agents have the same structural unit, except for the terminal groups, which are ethylene, octadecyl, or amino. Amino acids are hydrophilic; therefore, the Cu_2_O QDs decorated with 3‐aminopropyltriethoxysilane still showed strong hydrophilicity and were difficult to disperse in nonpolar solvent, potentially leading to a low‐quality Cu_2_O film. In contrast, modification with octadecyltriethoxysilane could enhance the hydrophobicity of Cu_2_O because of the hydrophobic octadecyl groups, which favor the dispersion of Cu_2_O QDs in nonpolar solvent. However, the charge mobility of the Cu_2_O film exhibited an obvious decline with octadecyltriethoxysilane modification due to its long carbon chain, which may impede hole transport in the Cu_2_O layer (Figure 2e). In summary, both the hydrophobicity and conductivity of silane coupling agents should be carefully considered to maintain the high dispersion and charge mobility of Cu_2_O QDs. Based on this principle, ethenyltriethyloxysilane was explored as the silane coupling agent due to its hydrophobic, short ethenyl group. The dispersion of Cu_2_O QDs was enhanced by the presence of ethenyltriethyloxysilane, and the modified Cu_2_O film still shows excellent hole mobility.

**Figure 2:**
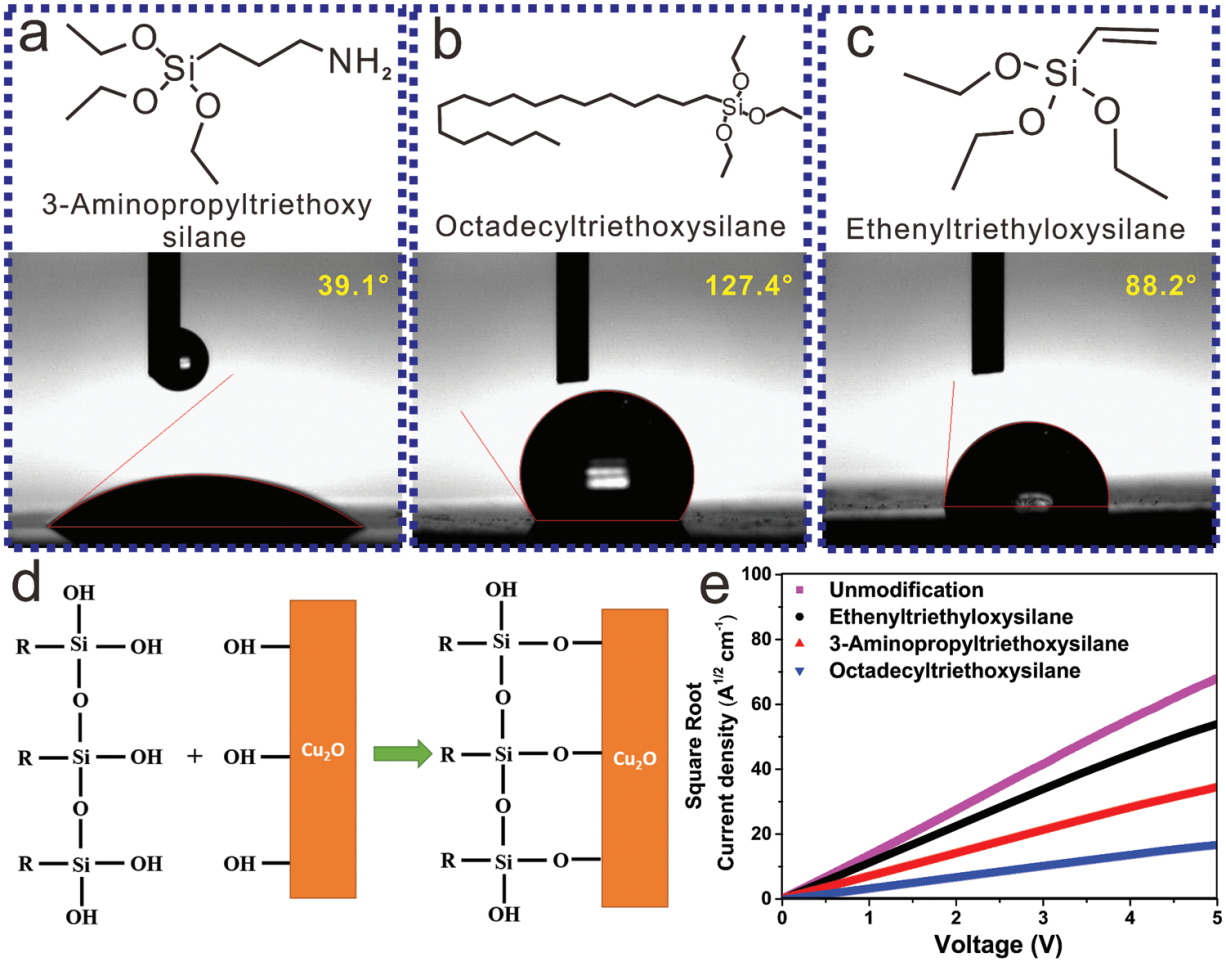
a–c) Structures of the three silane coupling agents used as the surface modification agent for Cu_2_O and contact angle measurements of the Cu_2_O QD films prepared with these silane coupling agents. d) The interface modification mechanism of the silane coupling agents on Cu_2_O QDs. e) SCLC measurement of the Cu_2_O layer.

**Figure** **3**a shows scanning electron microscopy (SEM) images of the synthesized small Cu_2_O QDs with a cubic morphology. The particles are highly uniform in size and shape. To maintain a clean surface, Cu_2_O QDs with an average diameter of 8−10 nm were synthesized in aqueous solution without the use of surfactant. The formation of such uniformly sized nanocubes is very important for their subsequent surface modification. More information about the Cu_2_O QDs was provided by transmission electron microscopy (TEM). Figure 3b shows typical TEM images of the Cu_2_O QDs. The size and morphology of the Cu_2_O particles were similar to those determined by SEM. A corresponding high‐resolution TEM image (Figure 3c) taken from a single nanocube gives lattice fringes with spacing that match the cube orientation and confirms that the (100) planes are aligned parallel to its {100} faces. Clear lattice fringes corresponding to the {110} plane of Cu_2_O were observed on the Cu_2_O nanocube, indicating high crystallinity of the nanoparticles. As shown in Figure 3d, the corresponding selected area electron diffraction (SAED) pattern shows obvious (110) and (101) diffraction spots for Cu_2_O, further indicating the formation of Cu_2_O. To determine the composition of the deposits, X‐ray diffraction (XRD) analysis was performed. Figure S2a (Supporting Information) shows an XRD spectrum of the Cu_2_O QDs. The diffraction peaks are identified as pure Cu_2_O according to JCPDS 99‐0041 data. An intense peak at 2θ = 36.4° corresponds to the (111) crystal plane. Other prominent peaks correspond to the (200), (220), and (311) planes (Figure S2, Supporting Information). As shown in Figure S2 (Supporting Information), there is no difference between the XRD patterns of unmodified and modified Cu_2_O, indicating that the crystalline structure of the Cu_2_O QDs did not change after surface modification with ethenyltriethyloxysilane. The change in surface composition was further verified by UV–vis spectroscopy (Figure S3, Supporting Information). The absorption spectra of unmodified and modified Cu_2_O show a typical absorption onset at 560 nm. This indicates that the absorption of the Cu_2_O QDs did not change after ethenyltriethyloxysilane modification, which is consistent with that concluded from XRD spectra. To further study the chemical modification of ethenyltriethyloxysilane on the surface of the Cu_2_O QDs, the differences between the Fourier transform infrared spectroscopy (FTIR) spectra of unmodified and modified Cu_2_O QDs were investigated, and the result is shown in Figure S4 (Supporting Information). Both the unmodified and modified Cu_2_O samples contain the same band at 627 cm^−1^, which is attributed to the stretching vibration of Cu(I)—O (Cu_2_O) and agrees with previous literature. For the unmodified Cu_2_O QDs, the band at 3415 cm^−1^ corresponds to the stretching vibration of —OH groups, and the bands at 1480 and 1380 cm^−1^ are assigned to —OH bending vibrations. These bands prove the presence of —OH groups on the surface of the unmodified Cu_2_O QDs.^43^ After surface modification, the bands belonging to —OH groups disappear, and bands for C—Si—O at 1407, 1045, and 950 cm^−1^ appear, indicating that the OH groups on the surface of Cu_2_O were replaced by silanes.^44^ Furthermore, Cu_2_O may strongly interact with silane, as demonstrated by the band at 765 cm^−1^ assigned to the vibrations of Si—O—Cu.^45^

**Figure 3:**
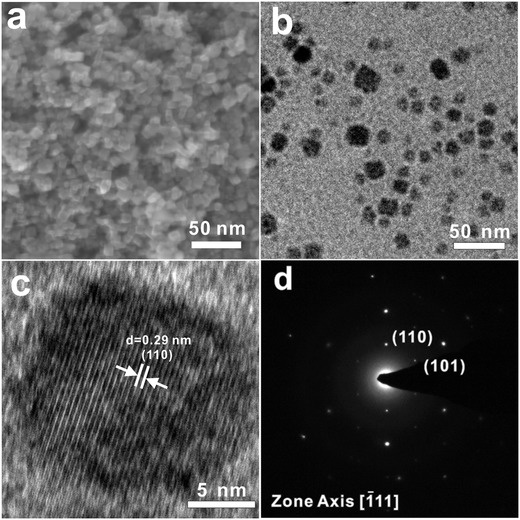
a,b) SEM and TEM images of Cu_2_O nanoparticles prepared by a simple solution process. c) HR‐TEM images of an individual Cu_2_O nanoparticle. d) The corresponding SAED pattern viewed along the [111] direction.

As shown in **Figure** **4**a, the as‐formed CsFAMA film exhibited full surface coverage and was composed of nanometer‐sized grains ranging from tens of nanometers to hundreds of nanometers in size. The crystallinity of the resulting CsFAMA film deposited on the FTO substrate was investigated by XRD spectroscopy (Figure S5, Supporting Information). The diffraction peaks at 14.10°, 20.01°, 23.48°, 24.50°, 28.43°, 31.88°, 40.67°, and 43.20° can be assigned to the (110), (112), (211), (202), (220), (310), (224), and (314) planes, respectively, of the perovskite tetragonal phase, indicating a fully formed perovskite structure.^46^ After deposition of the unmodified Cu_2_O layer on the perovskite film (Figure 4b), many holes and defects could be observed on the surface, attributed to the poor dispersity of unmodified Cu_2_O QDs in the 2‐methylanisolsolution. After surface modification, the modified Cu_2_O film appeared homogeneous, and the perovskite grains became invisible, suggesting that the perovskite film surface was completely covered by Cu_2_O QDs (Figure 4c). The cross‐sectional SEM image of the FTO/TiO_2_/perovskite/Cu_2_O/Au film (Figure 4d) indicates that the perovskite layer is very compact and has a thickness of ≈500 nm, and the Cu_2_O HTM layer has a thickness of ≈100 nm that uniformly covered the perovskite layer. An ≈30 nm compact TiO_2_ blocking layer was deposited on the FTO glass below the perovskite layer. The full coverage of the perovskite film by Cu_2_O and the prevention of direct contact between the Cu_2_O HTM layer and the bottom of the TiO_2_ layer by the compact perovskite layer should prohibit charge recombination. Finally, a 100 nm Au electrode was thermally evaporated on the top of the Cu_2_O layer to complete the device.

**Figure 4:**
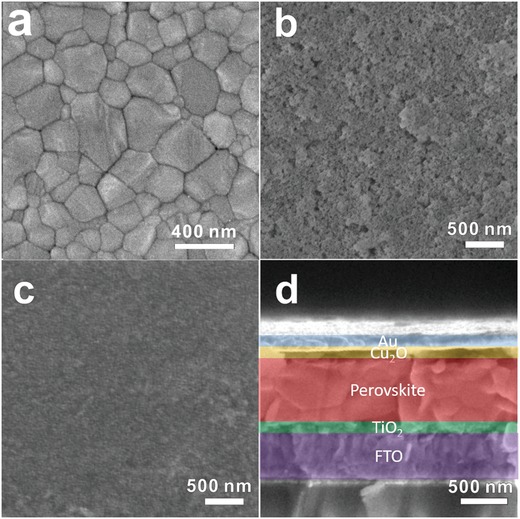
SEM images of a PSC based on Cu_2_O as the HTM: a–c) top view of the perovskite layer, unmodified Cu_2_O layer, and modified Cu_2_O layer surfaces, respectively. d) Cross section of the modified Cu_2_O‐based device with an Au coating.

**Figure** **5**a shows the current *J*–*V* curves of PSCs employing unmodified, modified Cu_2_O, and Spiro‐OMeTAD as the HTM under AM 1.5 G illumination at 100 mW cm^−2^. A device fabricated with unmodified Cu_2_O HTM exhibited an open‐circuit voltage (*V*_oc_) of 1.00 V, a short‐circuit current density (*J*_sc_) of 20.70 mA cm^−2^, and a fill factor (FF) of 57.5%, resulting in a best PCE of 11.9% in the reverse scanning direction. A device using modified Cu_2_O as the HTM displayed a *V*_oc_ of 1.15 V, a *J*_sc_ of 22.2 mA cm^−2^, and an FF of 74.2%, reaching a best PCE of 18.9%. The overall efficiency enhancement mainly resulted from the significant increase in the *V*_oc_, *J*_sc_, and FF values. Compared with the PCE (20.5%) obtained when the commonly used Spiro‐OMeTAD was employed as the HTM, the PCE of the modified Cu_2_O‐based device was comparable (18.9%) and was much higher than that of the unmodified Cu_2_O‐based device. To the best of our knowledge, the PCE of the PSC with modified Cu_2_O as the HTM represents the highest performance reported for a PSC with an n‐i‐p structure based on the Cu_2_O HTM. The device performance statistics for the devices based on unmodified Cu_2_O, modified Cu_2_O, and Spiro‐OMeTAD were obtained on the basis of 20 independent devices (Table S2, Supporting Information). The average PCE values follow the same trend as the highest values discussed above. The external quantum efficiency (EQE) spectra of the devices with different HTMs are plotted in Figure 5b. Compared with the spectrum of the device with unmodified Cu_2_O, the EQE spectra of the devices employing modified Cu_2_O and Spiro‐OMeTAD HTM are significantly higher in the range of 300–800 nm, especially in the longer wavelength region. The increased spectral response should be attributed to the improved charge collection in the presence of HTM. The EQE of the device using Cu_2_O HTM is lower than that of the device using Spiro‐OMeTAD HTM in the region from 350 to 670 nm. However, the EQE of the device employing Cu_2_O HTM exhibits a smaller decrease in the region from 650 to 750 nm than does the device employing Spiro‐OMeTAD HTM, which can be attributed to the smaller band gap of Cu_2_O. Notably, the Cu_2_O layer can improve the EQEs of PSCs in the long wavelength region because of the photocurrent originating from the Cu_2_O layer, which is similar to previous reports about the extension of the photoresponse toward longer wavelengths by CuInS_2_ and Cu_2_ZnSnS_4_ layers.^47^, ^48^ This indicates that Cu_2_O can potentially be used to expand the photoresponse of PSCs. As shown in Figure 5b, the integrated current values calculated by the EQE spectra for the devices using unmodified Cu_2_O, modified Cu_2_O, and Spiro‐OMeTAD are 18.9, 21.8, and 23.0 mA cm^−2^, respectively. The integrated current density from the EQE spectra for each device agrees with the current density obtained from the *J*–*V* curves. Furthermore, to determine the stabilized (scan‐speed‐independent) PCEs, the solar cells were probed at their maximum power point (MPP) under full‐sun illumination (Figure 5c). We recorded a stabilized output power corresponding to a PCE of 11.5%, 18.6%, and 20.0% for unmodified Cu_2_O, modified Cu_2_O, and Spiro‐OMeTAD‐based devices, respectively, in close agreement with the *J*–*V* measurements.

**Figure 5:**
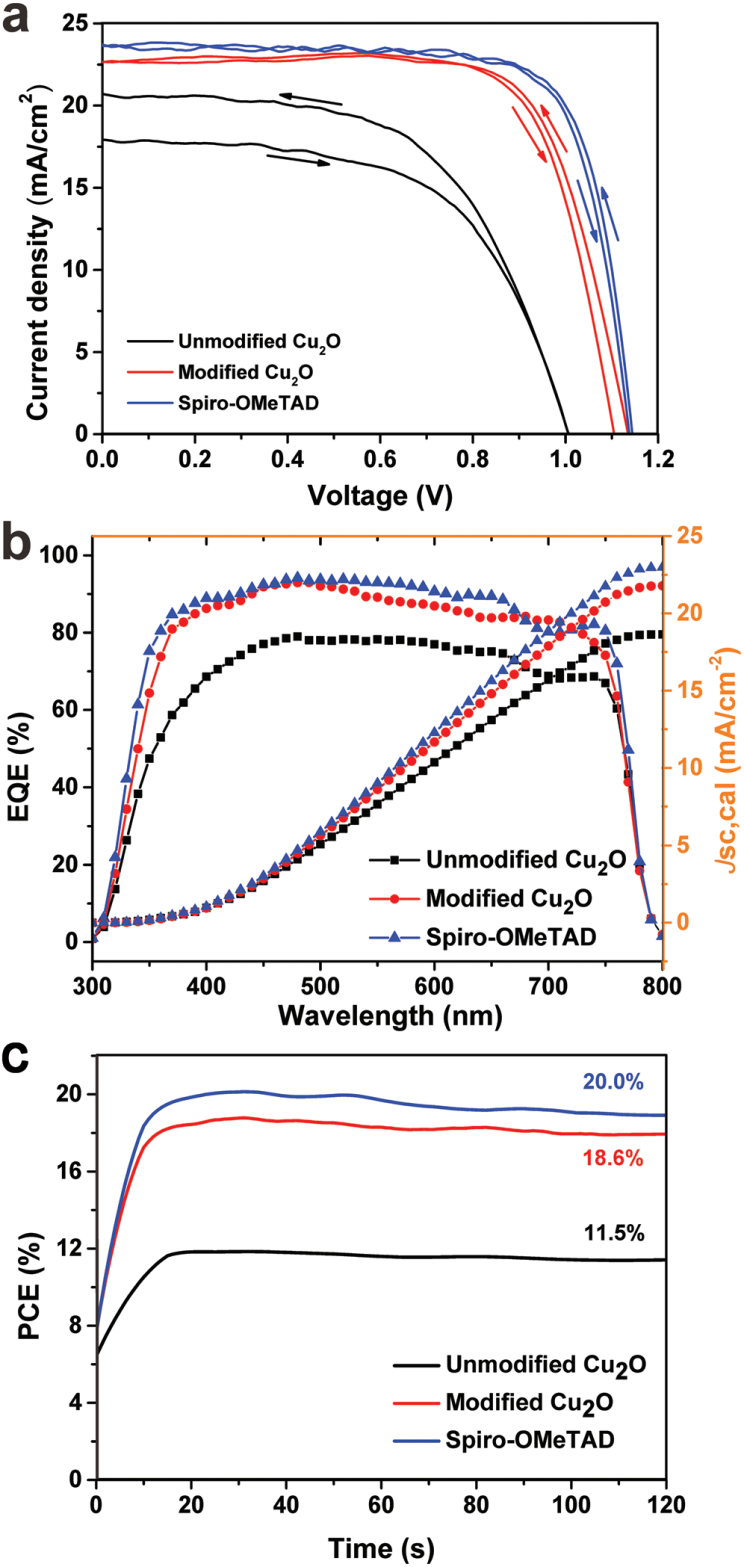
a) *J–V* curve of the best‐performing devices based on Cu_2_O and on Spiro‐OMeTAD HTM at a 0.01 V s^−1^ scanning rate in reverse and forward directions. b) Corresponding EQEs of the PSCs. c) MPP tracking for 120 s to yield a stabilized efficiency for unmodified Cu_2_O, modified Cu_2_O, and Spiro‐OMeTAD‐based devices.

We investigated the charge‐carrier dynamics in pristine and HTM‐containing perovskite films using steady‐state photoluminescence (SSPL) and time‐resolved PL (TRPL) spectra.^49^ As shown in **Figure** **6**a, the pristine perovskite film exhibited an intense PL emission centered at ≈780 nm with a linewidth of 60 nm. The perovskite PL quantum yield of the film with modified Cu_2_O QDs was largely reduced compared to that of the film with unmodified Cu_2_O QDs, indicating a significantly enhanced charge carrier extraction arising from the addition of modified Cu_2_O QDs. Furthermore, the dynamics of charge carriers were quantitatively studied by TRPL measurements. As shown in Figure 6b, the long PL decay lifetime of the pristine perovskite film was determined to be 50 ns, showing high electronic quality. The PL in the perovskite/modified Cu_2_O film showed significant decay that was much faster than that in the perovskite/unmodified Cu_2_O film, indicating faster charge transfer from perovskite to the HTM.^50^ These results indicate that the perovskite films with modified Cu_2_O QDs can extract and transport holes more efficiently than films with unmodified Cu_2_O QDs, which is consistent with the results for the performance of the PSC devices. To further clarify the effect of Cu_2_O QDs, electrochemical impedance spectroscopy (EIS) was carried out to study the photogenerated charge recombination processes in PSCs. As shown in Figure 6c, the arc in the high‐frequency range, which is associated with the recombination resistance of perovskite/modified Cu_2_O QDs, is drastically larger than that of perovskite/unmodified Cu_2_O QDs, further implying reduced recombination losses.^48^

**Figure 6:**
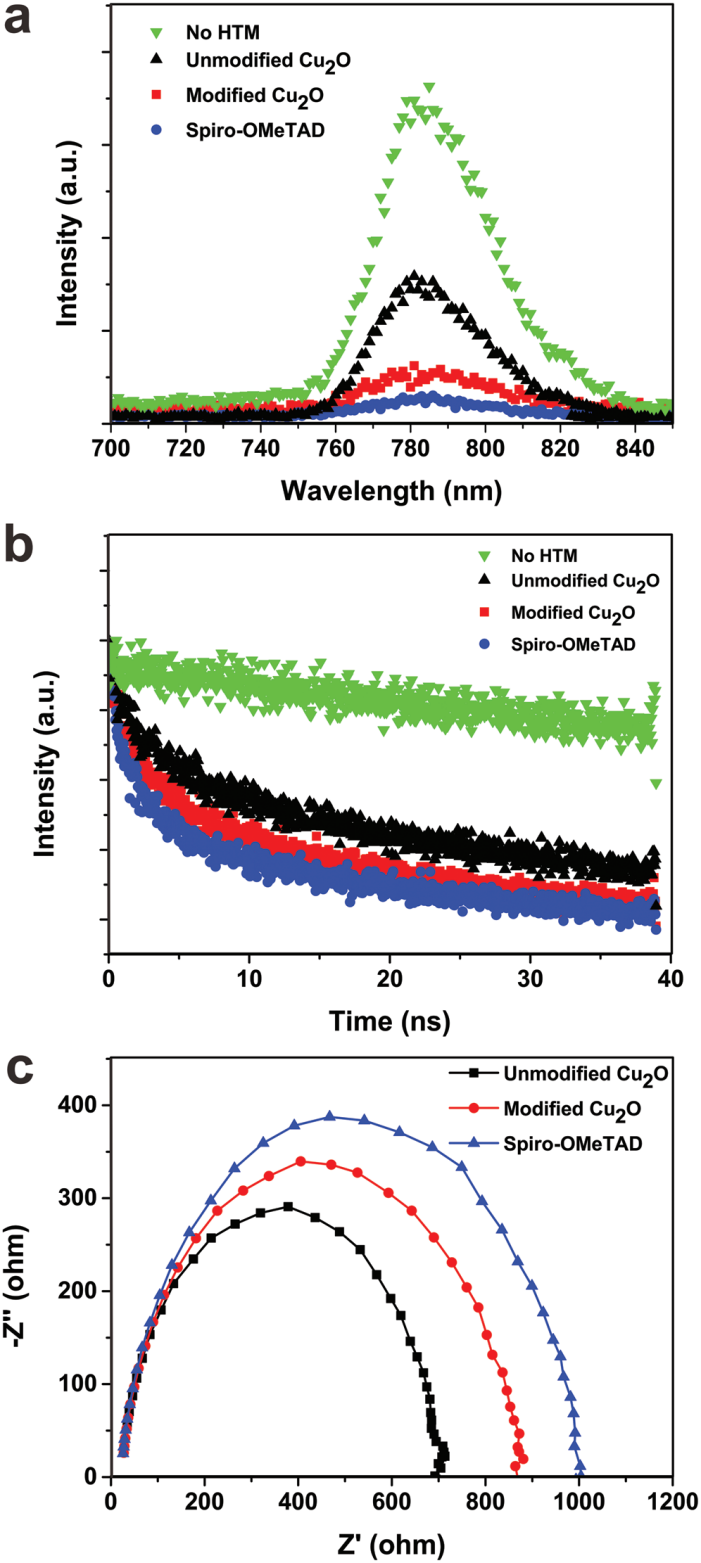
a) SSPL for glass/perovskite/HTM and b) TRPL for glass/perovskite/HTM. c) Nyquist plots of PSC devices with different HTMs.

The long‐term stability of PSCs is very important for practical applications. Although Spiro‐OMeTAD has been widely used as an HTM in high‐performance PSCs, the use of hygroscopic lithium salt doping is unfavorable for device stability. In this respect, dopant‐free Cu_2_O that is stable under ambient conditions is beneficial for solar cell applications. To verify this applicability, the air stability of PSCs without encapsulation was investigated, as shown in **Figure** **7**a. When stored in air with a relative humidity of ≈30%, the devices with unmodified Cu_2_O retained 80% of their original efficiency after 30 d, while the cells with Spiro‐OMeTAD retained only 50% after 30 d. This result indicates that such a hydroscopic ion additive (Li‐TFSL) should be avoided in practical applications because of its negative influence on device stability. Furthermore, the stability of PSCs can be further enhanced by surface modification. The modified Cu_2_O‐based PSC maintained over 90% of its original PCE after 30 d, exhibiting excellent ambient air stability. The difference in device stability resulted from different hydrophobicities of the HTMs. The modified Cu_2_O film shows a water contact angle of 88.2° (Figure 7b), indicating that the hydrophobic HTM can efficiently prevent water penetration into the perovskite layer. In comparison, the unmodified Cu_2_O film exhibits a smaller water contact angle of ≈24.7°, indicating an increased affinity for water caused by the surface hydroxyl groups.

**Figure 7:**
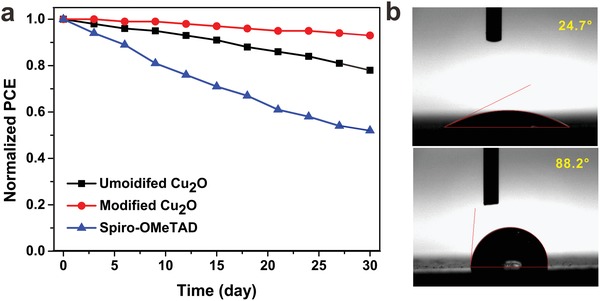
a) Device performance durability of PSCs with unmodified Cu_2_O, modified Cu_2_O, and Spiro‐OMeTAD in ambient air for 30 d. b) Water contact angles of the unmodified Cu_2_O and modified Cu_2_O films.

In summary, we have demonstrated a surface modification strategy for the dispersal of inorganic Cu_2_O QDs in a nonpolar solvent, which achieves the direct deposition of an inorganic HTM on the top of a perovskite layer in a mesoporous n‐i‐p structure. The modified Cu_2_O‐based device exhibits an enhanced PCE of 18.9% compared with that of the unmodified Cu_2_O‐based device (11.9%), which could be comparable to that of Spiro‐based devices. In this work, we achieved the record efficiency Cu_2_O‐based perovskite solar cell in n‐i‐p structure by using all inorganic interfacial layer. Owing to the dopant‐free technology and hydrophobic surface of Cu_2_O HTM, the prepared cells show excellent long‐term durability over more than 1 month, while the performance of Spiro‐based devices quickly declined and had completely deteriorated by 30 d. This proposed new mechanism and strategy of utilizing surface modification to enhance the dispersion of inorganic HTMs in nonpolar solvents has extremely wide applicability to all available inorganic materials to achieve the best performance. Furthermore, the high PCE and remarkable stability displayed by the PSC employing all‐inorganic charge extraction layers, i.e., mesoporous TiO_2_ and Cu_2_O, make this strategy very promising for practical applications. Given the above uniqueness, this novel and as‐established approach could be universally applicable and practical for the considerable enhancement of PSCs, which may be also of significance in the field of HTM surface modification for other type solar cells and electronic devices.

## Experimental Section

*Materials*: Mesoporous TiO_2_ paste (Dyesol), formamidinium iodide (FAI), methylammonium bromide (MABr), and lead bromide (PbBr_2_) were purchased from Xi'an Polymer Light Technology Corp. Spiro‐OMeTAD was purchased from Lumtec Corp. Sodium hydroxide (NaOH, 98%), L‐(+)‐ascorbic acid (AA, 99.7%), copper(II) sulfate pentahydrate (CuSO_4_·5H_2_O, 99%), and lead iodide (PbI_2_, 99.9985%) were purchased from Alfa Aesar. All of the other salts and anhydrous solvents, including Li‐TFSI salt, titanium diisopropoxide bis(acetylacetonate) (75 wt% in isopropyl alcohol), *N,N*‐dimethylformamide (DMF), ethanol, isopropyl alcohol, tert‐butylpyridine (tBP), chlorobenzene, acetonitrile, 1‐butanol, and dimethyl sulfoxide (DMSO), were purchased from Sigma‐Aldrich. Ethenyltriethyloxysilane, octadecyltriethoxysilane, and 3‐aminopropyltriethoxysilane were purchased from Shanghai Macklin Biochemical Co., Ltd. All of the above chemical products were used directly without further purification or treatment.

*Synthesis of Cu_2_O QDs*: Deionized water (9 mL) and 100 µL of 0.1 m CuSO_4_ solution were added to a sample vial. The vials were kept in a water bath at 35 °C throughout the particle synthesis. Next, 350 µL of 1.0 m NaOH solution was introduced into the sample vial with vigorous stirring. Upon the addition of 500 µL of 0.2 m AA to the above solution, the solution turned bright yellow immediately. The solution was stirred for 10 min in a water bath to allow crystal growth and then centrifuged at 8500 rpm for 10 min. After the supernatant was decanted, the precipitate was centrifuged and washed three times with 40 mL of a 1:1 volume ratio of water and ethanol to remove unreacted chemicals. After a final washing with 30 mL of ethanol, the precipitate was dispersed in 2 mL of ethanol before storage and analysis.

*Surface Modification of Cu_2_O QDs*: The self‐assembly monolayer of silane molecules was formed as follows: 20 mg of Cu_2_O QDs was diluted in 10 mL of deionized water. The silane coupling agent was added in different molar ratios (20, 10, and 5 × 10^−3^ m) to determine the optimal concentration. The mixed solution was stirred for 120 min to allow the alkylalkoxysilane molecules to self‐assemble on the Cu_2_O surface. Subsequently, the Cu_2_O QDs were washed with ethanol three times to remove the physically absorbed silane coupling agents on the surface of the Cu_2_O QDs. Other silane coupling agent modifications were prepared in a similar process.

*Device Fabrication*: Pieces of FTO glass (Nippon Sheet Glass) were cleaned with detergent, deionized water, and acetone and sonicated with ethanol in an ultrasonic bath for 30 min. Then, the FTO glass was treated in a UV cleaner for 30 min. The pieces of cleaned FTO glass were coated with 0.15 m titanium diisopropoxide bis(acetylacetonate) in 1‐butanol by the spin‐coating method at 2000 rpm for 60 s, followed by heating at 125 °C for 5 min. The resulting films were cooled to room temperature, and a 0.15 m titanium diisopropoxide bis(acetylacetonate) solution in 1‐butanol was spin‐coated on the surface again to create pin hole‐free dense TiO_2_ films. Then, the substrates were calcined in a box furnace at 450 °C for 30 min. An mp‐TiO_2_ layer was deposited on the dense TiO_2_ layer by spin‐coating an ethanolic TiO_2_ solution containing 14.3 wt% TiO_2_ paste at 4000 rpm for 20 s, and the film was then calcined at 500 °C for 0.5 h. To achieve higher efficiency and stability, a multiple‐component perovskite film was selected as light harvest layer as reported by the previous reference.^51^ The precursor solution of mixed perovskite consisted of 172 mg of FAI, 507 mg of PbI_2_, 22.4 mg of MABr, and 73.4 mg of PbBr_2_ dissolved in 1 mL of a mixed solvent of DMF and DMSO with a volume ratio of 9:1. Then, a stock solution of 1.5 m CsI in DMSO was added to the mixed perovskite precursor to obtain the Cs_0.05_FA_0.81_MA_0.14_PbI_2.55_Br_0.45_ precursor solution. The perovskite films were deposited onto the TiO_2_ substrates via a two‐step spin‐coating procedure. The first step was performed at 1000 rpm for 10 s with an acceleration of 500 rpm s^−1^. The second step was performed at 4000 rpm for 35 s with a ramp‐up of 2000 rpm s^−1^. Chlorobenzene (≈100 µL) was quickly dropped onto the spinning substrate during the second spin‐coating step at 15 s before the end of the procedure. Afterward, the as‐prepared films were heated at 100 °C for ≈2 h until their color changed to dark red. The hole‐transport layer was prepared by spin‐coating an HTM dispersion, which was prepared by dispersing Cu_2_O QDs in 2‐methylanisole (20 mg mL^−1^), at 4000 rpm for 30 s. The Spiro‐OMeTAD solution was prepared by dissolving Spiro‐OMeTAD at 65 mg mL^−1^ in a chlorobenzene solution that contained 20 µL mL^−1^ tBP and 70 µL mL^−1^ Li‐TFSI salt (170 mg mL^−1^ in acetonitrile). Finally, a 100 nm thick Au layer was thermally evaporated at a rate of ≈0.05 nm s^−1^ under a vacuum of 4 × 10^−5^ Torr to complete the device fabrication.

*Measurements and Characterization*: UV–vis absorption spectra were recorded using a Perkin Elmer Lambda 950 UV–vis–NIR spectrometer. The PL spectra were acquired using a Horiba Fluoromax‐4 spectrofluorometer. The TRPL was measured with an Edinburgh Instrument FLS920 using a 375 nm laser derived from an Nd:YAG laser as the excitation source. FTIR spectral data for the devices were collected in the 700–3600 cm^−1^ range by using a Nicolet NEXUS 870 FT‐IR spectrometer. Drop shape analysis was performed using a drop shape analyzer (AST VCA Optima XE) with 18 MΩ water and a 4 µL dispense volume. Drops were measured at six different places across the substrate at room temperature 25 °C. XRD data were collected using a Bruker D8 Discover X‐ray diffractometer with Cu Kα radiation (1.54 Å) at 40 kV and 25 mA and with an Hi‐Star 2D area detector. TEM images were obtained using a Tecnai F30 microscope at 300 kV. The morphology and structure of the films were characterized by field emission scanning electron microscopy (ZEISS Merlin) at a 5 kV acceleration voltage. EIS data were recorded by an electrochemical workstation (CHI660, China) in a frequency range of 1 Hz–1 MHz applied in the dark. Photocurrent density–voltage (*J*–*V*) curves were measured under AM 1.5G one‐sun illumination (100 mW cm^−2^) with a solar simulator (Enlitech SS‐F7‐3A) equipped with a 300 W xenon lamp and a Keithley 2400 source meter. The light intensity was adjusted by an National Renewable Energy Laboratory (NERL)‐calibrated Si solar cell. During measurement, the cell was covered with a mask possessing an aperture of 0.1 cm^2^. The EQE was measured with an EQE system (Enlitech QE‐R) containing a xenon lamp, monochromator, Si detector, and dual‐channel power meter.

## Conflict of Interest

The authors declare no conflict of interest.

## Supporting information

Supplementary
