# Supplementary material for: Hydrophobic Cu2O Quantum Dots Enabled by Surfactant Modification as Top Hole‐Transport Materials for Efficient Perovskite Solar Cells
Source: Adv Sci (Weinh). 2019 Feb 7;6(7):1801169. doi: 10.1002/advs.201801169 (PMC6446601; doi:10.1002/advs.201801169)
Supplement: Supplementary file 1 — Supplementary [file ADVS-6-1801169-s001.pdf]

Copyright WILEY-VCH Verlag GmbH & Co. KGaA, 69469 Weinheim, Germany, 2019.

ADVANCED SCIENCE
Open Access

Supporting Information

for *Adv. Sci.,* DOI: 10.1002/advs.201801169

Hydrophobic Cu_2O Quantum Dots Enabled by Surfactant Modification as Top Hole-Transport Materials for Efficient Perovskite Solar Cells

*Chang Liu, Xianyong Zhou, Shuming Chen, Xingzhong Zhao, Songyuan Dai, and Baomin Xu**

Copyright WILEY-VCH Verlag GmbH & Co. KGaA, 69469 Weinheim, Germany, 2019.

Supporting Information

Hydrophobic Cu_2O Quantum Dots Enabled by Surfactant Modification as Top Hole-Transport Materials for Efficient Perovskite Solar Cells

Chang Liu, Xianyong Zhou, Shuming Chen, Xingzhong Zhao, Songyuan Dai, and Baomin Xu*

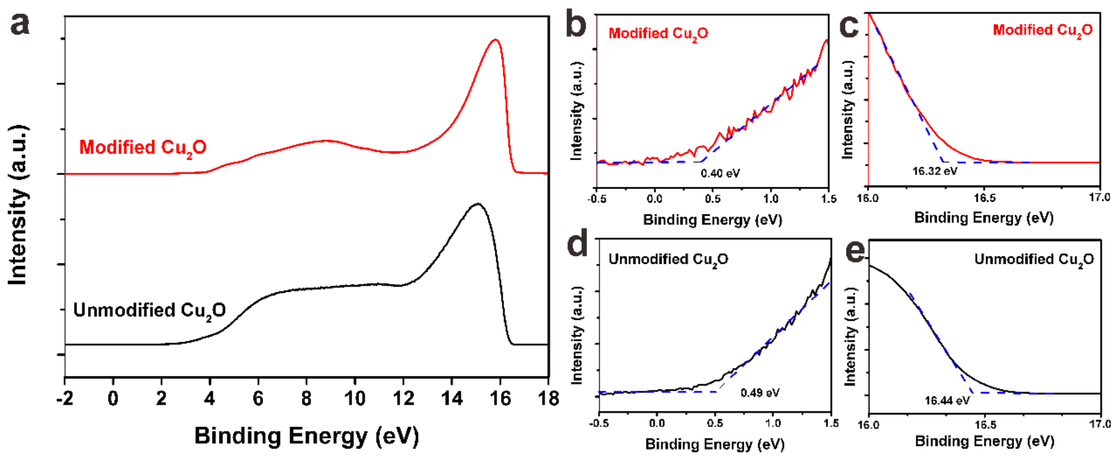

Figure S1. (a) UPS spectra of the unmodified Cu_2O and modified Cu_2O used for the determination of the valence band maximum energies and semiconductor work function. (b-e) Expanded UPS spectra.

Table S1. Photoemission parameters obtained from UPS measurement and band energy level of unmodified Cu_2O and modified Cu_2O.

Sample	$E_{valence}$ [eV]	High B.E. [eV]	WF [eV]	E_{VB} [eV]	E_{CB} [eV]
Unmodified Cu_2O	0.49	16.44	4.76	5.25	3.16
Modified Cu_2O	0.40	16.32	4.88	5.28	3.19

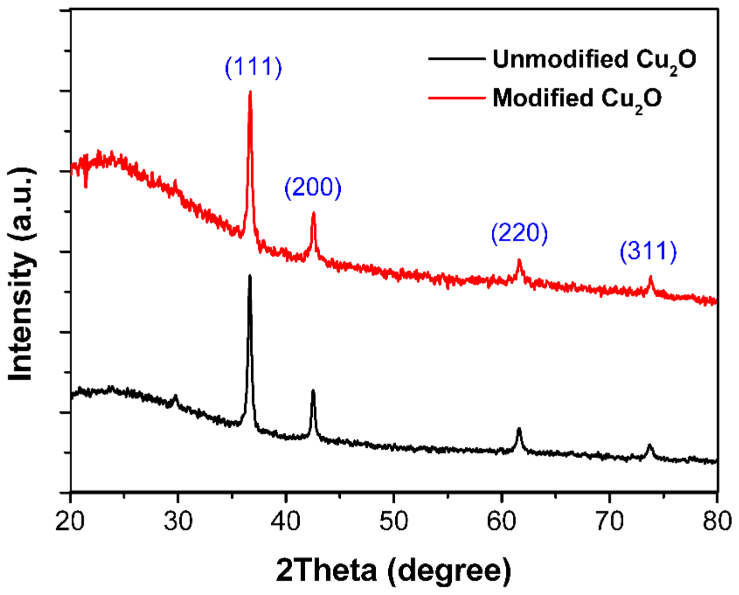

Figure S2. The XRD spectra of Cu_2O QDs with and without Ethenyltriethyloxy-silane modification.

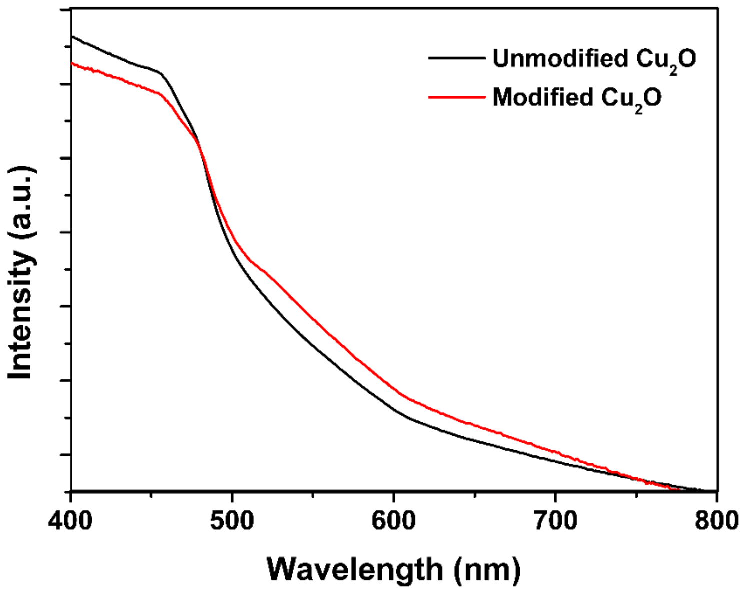

Figure S3. The UV-VIS spectra of Cu_2O QDs with and without Ethenyltriethyloxy-silane modification.

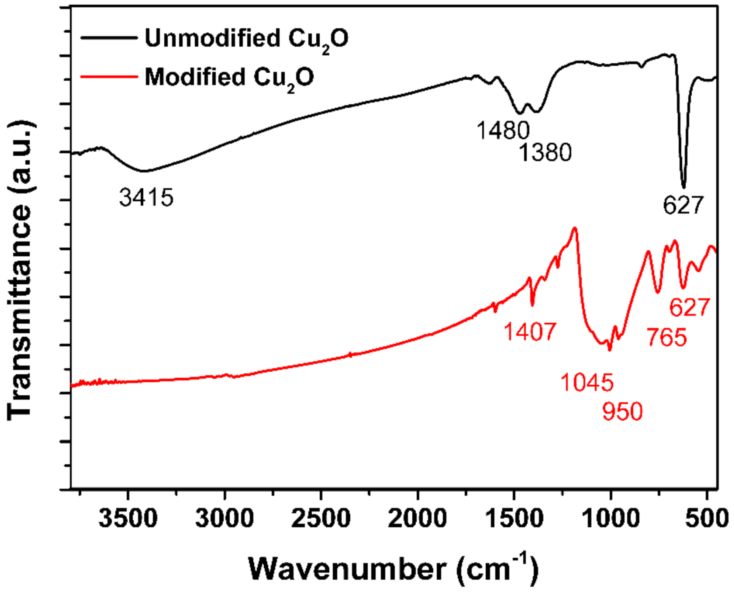

Figure S4. The FTIR spectra of Cu_2O QDs with and without Ethenyltriethyloxy-silane modification.

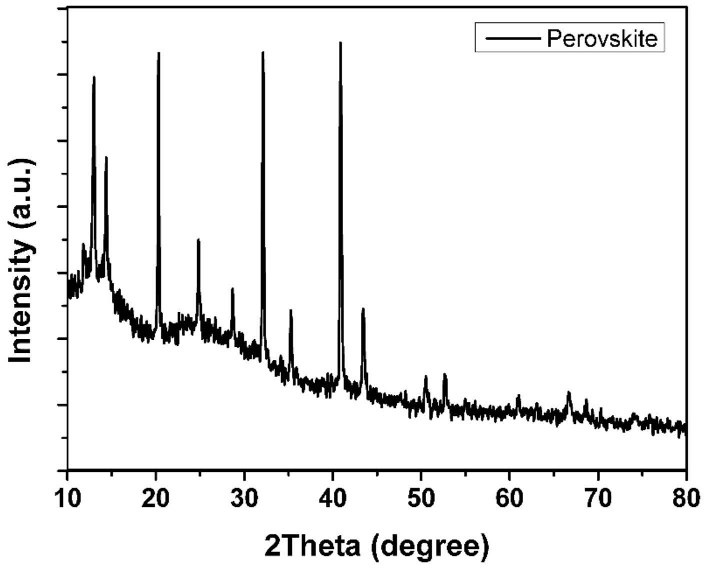

Figure S5. The XRD spectrum of perovskite film.

Table S2. Photovoltaic properties of the optimized PSCs based on unmodified Cu_2O, modified Cu_2O and Spiro-OMeTAD.

HTM layer	V_{OC} [V]	J_{SC} [mA cm^{-2}]	FF [%]	PCE [%]

Unmodified Cu_2O	0.99±0.04	17.52±0.34	58.46±4.65	10.9±1.1
Modified Cu_2O	1.12±0.03	22.17±0.42	70.46±4.32	18.2±0.7
Spiro-OMeTAD	1.13±0.02	23.25±0.29	70.41±2.37	20.2±0.4
